# Effects of cyclophosphamide on pulmonary function in patients with scleroderma and interstitial lung disease: a systematic review and meta-analysis of randomized controlled trials and observational prospective cohort studies

**DOI:** 10.1186/ar2534

**Published:** 2008-10-20

**Authors:** Carlotta Nannini, Colin P West, Patricia J Erwin, Eric L Matteson

**Affiliations:** 1 Division of Rheumatology, Mayo Clinic College of Medicine, 200 First Street SW, Rochester, MN 55905, USA; 2 Division of General Internal Medicine, Mayo Clinic College of Medicine, 200 First Street SW, Rochester, MN 55905, USA; 3 Division of Biostatistics, Mayo Clinic College of Medicine, 200 First Street SW, Rochester, MN 55905, USA; 4 Medical Library, Mayo Clinic College of Medicine, 200 First Street SW, Rochester, MN 55905, USA

## Abstract

**Introduction:**

The purpose of the present study was to systematically review the effect of cyclophosphamide treatment on pulmonary function in patients with systemic sclerosis and interstitial lung disease.

**Methods:**

The primary outcomes were the mean change in forced vital capacity and in diffusing capacity for carbon monoxide after 12 months of therapy in patients treated with cyclophosphamide.

**Results:**

Three randomized clinical trials and six prospective observational studies were included for analysis. In the pooled analysis, the forced vital capacity and the diffusing capacity for carbon monoxide predicted values after 12 months of therapy were essentially unchanged, with mean changes of 2.83% (95% confidence interval = 0.35 to 5.31) and 4.56% (95% confidence interval = -0.21 to 9.33), respectively.

**Conclusions:**

Cyclophosphamide treatment in patients with systemic sclerosis-related interstitial lung disease does not result in clinically significant improvement of pulmonary function.

## Introduction

Scleroderma (systemic sclerosis (SSc)) is an autoimmune connective tissue disorder characterized by microvascular injury, excessive fibrosis of the skin and distinctive visceral changes that can involve the lungs, heart, kidneys and gastrointestinal tract [1]. Interstitial lung disease (ILD) occurs in patients who have CREST (Calcinosis, Raynaud, ESophagitis, Telangiectases), limited cutaneous systemic sclerosis-lcSSc and diffuse cutaneous scleroderma (dcSSc), but it is somewhat more common in patients who have diffuse disease [2,3]. The ILD that occurs in scleroderma patients includes a number of entities, as summarized in Table 1[4]. The prevalence of ILD in scleroderma varies from 25% to 90% depending on the ethnic background of the patients studied and on the method used to detect the ILD [5].

**Table 1:** Interstitial lung disease entities associated with systemic sclerosis

Pulmonary fibrosis
- Nonspecific interstitial pneumonia (this is a subtype of fibrosis)
- Usual interstitial pneumonia (this is a suntype of fibrosis)
Fibrosing alveolitis
Diffuse alveolar damage
Cryptogenetic organizing pneumonia

Pulmonary function tests with evaluation of the forced vital capacity (FVC), the total lung capacity and the diffusing lung capacity of carbon monoxide (DLCO), chest radiography and high-resolution computed tomography are common clinical tests used to evaluate lung disease in scleroderma. Imaging reveals fibrotic changes of lung parenchyma. Previous research has found pulmonary function tests to reveal a restrictive pattern in 23% of patients with limited disease, and found 40% of patients with diffuse disease to have pulmonary fibrosis [4,5]. ILD as assessed by chest radiography has been reported in 33% of patients with limited scleroderma and in 40% of patients with diffuse SSc [5]. High-resolution computed tomography detects ILD changes in 90% to 100% of SSc patients [2,5].

ILD is associated with increased mortality in patients who have SSc. The greatest loss of lung volume occurs within the first 2 years of the disease, and pulmonary-related deaths occur with greater frequency in the second 5 years from disease onset [5]. Patients with severe lung involvement (defined as FVC < 55% and DLCO < 40% of predicted) have a worse prognosis, with a mortality of 42% within 10 years of the onset of disease [5].

A number of agents have been evaluated for treatment of SSc-related ILD but none have proven effective in altering the disease course. Cyclophosphamide (CYC) is a cytotoxic immunosuppressive agent that suppresses lymphokine production and modulates lymphocyte function by alkylating various cellular constituents and depressing the inflammatory response. Of all the drugs studied for the treatment of SSc-related ILD, only CYC has shown much promise of benefit in slowing down the decrease in, or even improving, lung function and survival [1]. Retrospective studies, pilot studies, and open-label clinical trials support the effectiveness of CYC therapy in preventing a decline in lung function and premature death in patients with SSc and ILD.

Despite these individual study results, previous systematic reviews of retrospective studies of the CYC effect in SSc lung disease have yielded conflicting results, suggesting either some or no benefit of this agent [6,7]. To determine the possible benefit of CYC as management for SSc-related ILD, we examined the benefit of CYC on lung function as measured by pulmonary function tests by conducting a systematic review and meta-analysis of randomized clinical trials and prospective observational studies in patients with SSc treated with CYC.

## Materials and methods

The study selection, assessment of eligibility criteria, data extraction and statistical analysis were performed based on a prespecified protocol according to the Cochrane Collaboration guidelines [8]. The present article has been prepared in accordance with the QUOROM statement [9]. An expert medical librarian searched Ovid EMBASE, Ovid MEDLINE, and the Ovid Cochrane Library from 1986 to 2008 using the terms *systemic scleroderma*, *autoimmune diseases*, *cyclophosphamide*, *immunosuppressive therapies*, *interstitial lung disease*, *randomized controlled trials*, *observational studies*, *multicenter studies*, *clinical trials phase II*, *clinical trials phase III*, and *clinical trials phase IV*.

To locate unpublished trials, we searched the electronic abstract databases of the annual scientific meetings of the European League Against Rheumatism, the American College of Rheumatology and the American Thoracic Society, from the approval of CYC as a treatment for autoimmune disease in 1986 to the present. No restriction for language was used.

Assessment of eligibility criteria for inclusion or exclusion and extraction of outcome variables was performed independently by two investigators (CN and ELM) with an intraobserver agreement kappa statistic of 1.

### Selection and outcomes

We selected randomized clinical trials [1,10,11] and prospective observational studies [12-18] that included patients classified as having limited and/or diffuse SSc according to the American College of Rheumatology criteria [19] and a diagnosis of ILD [20] treated with oral or intravenous CYC. The dose of CYC administered differed across the various cohorts of patients. Some studies expressed the CYC dose in milligrams per kilogram per day and others in milligrams per square meter of body surface. The oral dose of CYC ranged from 1 mg/kg/day to 2.5 mg/kg/day, and the intravenous dose of CYC ranged from 500 mg/m^2 ^to 750 mg/m^2 ^– except for one study in which 900 mg/kg/day intravenous CYC was administered (Tables 2 and 3).

**Table 2:** Randomized clinical trial study characteristics

Study	Number of patients	Mean age (years)	Outcome measure^a^	CYC treatment	Placebo/alternative treatment	Corticosteroid	Length of follow-up (months)
Hoyles and colleagues [10]	45	55	FVC, 80.1 ± 10.3	Intravenous, 600 mg/m^2 ^monthly	Placebo	Prednisone 20 mg alternate days	12
			DLCO, 52.9 ± 1.6				
Nadashkevich and colleagues [11]	60	38 to 36	FVC, 90.3 ± 1.9	Oral, 2 mg/kg/day monthly	AZA 2.5 mg/kg	Prednisolone 15 mg/day	12
			DLCO, 83.5 ± 1.6				
Tashkin and colleagues [1]	158	47.9 ± 1.0	FVC, 67.6 ± 1.3	Oral, 1 mg/kg/day	Placebo	None	12
			DLCO, 47.2 ± 1.6				

Data presented as mean ± standard deviation. AZA, azathioprine; CYC, cyclophosphamide; DLCO, diffusing capacity for carbon monoxide; FVC, forced vital capacity. ^a^Percentage predicted value at baseline.

**Table 3:** Observational study characteristics

Study	Number of patients	Mean age (years)	Outcome measure^a^	CYC treatment	Corticosteroid	Length of follow-up (months)
Airò and colleagues [15]	13	48	FVC, 74	Intravenous, 750 mg/m^2 ^every 3 weeks	Methylprednisolone 125 mg every 3 weeks	18
			DLCO, 41			
Beretta and colleagues [14]	33	49.7 ± 10.4	DLCO, 48.8 ± 13.5	Oral, 2 mg/kg/day	Prednisone 25 mg/day in the first 3 months, 5 mg for 9 months	12
Davas and colleagues, pulse CYC [12]	8	NA	FVC, 86.1	Intravenous, 750 mg/m^2 ^monthly	Prednisone 10 mg/day	12
			DLCO, 60			
Davas and colleagues, oral CYC [12]	8	NA	FVC, 73.2	Oral, 2 to 2.5 mg/kg/day	Prednisone 10 mg/day	12
			DLCO, 59.9			
Pakas and colleagues, low-dose prednisone cohort [16]	12	48.6 ± 12.3	FVC, 54.8	Intravenous, 900 mg/kg (mean value)	Prednisone low dose, <10 mg/day	12
			DLCO, 38.2			
Pakas and colleagues, high-dose prednisone cohort [16]	16	48.6 ± 12.3	FVC, 57.5	Intravenous, 900 mg/kg (mean value)	Prednisone: high dose, 1 mg/kg/day for 4 weeks	12
			DLCO, 48.3			
Silver and colleagues [17]	14	46.4 ± 2.4	FVC, 51.4 ± 2.5	Oral, 1 to 2 mg/kg/day	Prednisone 7.7 ± 1.2 mg/day (in 10 patients)	24
			DLCO, 54.5 ± 7.4			
Valentini and colleagues [18]	13	37.4	DLCO, 58.5	Intravenous, 500 mg/m^2 ^on day 1, day 8 and day 15, and every 4 weeks	Low dose corticosteroids (dose not specified)	12

Data presented as mean ± standard deviation. AZA, azathioprine; CYC, cyclophosphamide; DLCO, diffusing capacity for carbon monoxide; FVC, forced vital capacity; NA, not available. ^a^Percentage predicted value at baseline.

In the randomized clinical trials, patients were randomly allocated to receive treatment with CYC versus placebo [1,10] or versus azathioprine [11] for at least 12 months. In the observational prospective studies, scleroderma patients were treated with CYC for at least 12 months, and were evaluated at baseline and after 12 months of therapy. Corticosteroid treatment was permitted in both the randomized clinical trials and observational studies.

A clinically important change between two groups of treatment (CYC versus non-CYC) has been previously reported as an improvement ≥ 10% of the predicted value at 12 months or from the baseline value of FVC or DLCO [12,13]; we adopted this standard.

### Data abstraction and study validity

Data were abstracted for the difference in FVC and DLCO predicted values between baseline and 12 months of therapy. In these DLCO studies, the single-breath diffusing capacity was assessed by a carbon monoxide/helium gas mixture and was corrected for hemoglobin. The FVC was measured by spirometry using flow-volume loops [21]. Results are expressed as a percentage of the normal predicted values based on the patient's sex, age and height.

The methodological features of all randomized clinical trials most relevant to the prevention of bias (including the Jadad criteria of randomization, blinding and completeness of follow-up and outcome assessment [22]) were evaluated by two assessors (CN and ELM) independently, with disagreement resolved by consensus (see Additional file 1). Validity of observational studies was assessed following the Newcastle-Ottawa quality assessment scale for cohort studies (see Additional file 2) [23].

### Statistical analysis

Pre-post comparisons were made using paired *t *tests. Two observational studies had lengths of follow-up of 18 months [15] and of 24 months [17]; the FVC and DLCO values in these studies 12 months after CYC introduction were used. Dichotomous variables were compared using chi-square tests. Adverse event rates (occurrence of infections that required antibiotic therapy, hemorrhagic cystitis, hematuria and hospitalization) were calculated using relative risks for the randomized control trials representing the risk of an adverse event occurring in the CYC group compared with in the non-CYC group.

Two of the three randomized clinical trials reported the FVC and DLCO value at baseline and after 12 months in the CYC group but did not report standard errors [8,9]. The authors were contacted but were unable to provide standard error data. We therefore imputed the mean value of the standard errors of the other studies, and performed sensitivity analyses across the range of reported standard errors of these studies.

We used a random-effects model assessing the weighted mean difference in the meta-analysis. The overall pooled analysis included the mean changes of the FVC and the DLCO after 12 months of therapy obtained from the observational studies and from the CYC experimental arm of the randomized clinical trials. Additionally, we performed a meta-analysis of the randomized controlled trial results comparing CYC treatment with control treatments. Using a test of interaction, we performed a subgroup analysis of the change in FVC and DLCO values from baseline to 12 months in studies using oral administration of CYC versus those studies with intravenous administration. Analysis was conducted using Review Manager Version 4.2 (The Cochrane Collaboration^®^, Software Update, Oxford, UK).

## Results

**Figure 1:**
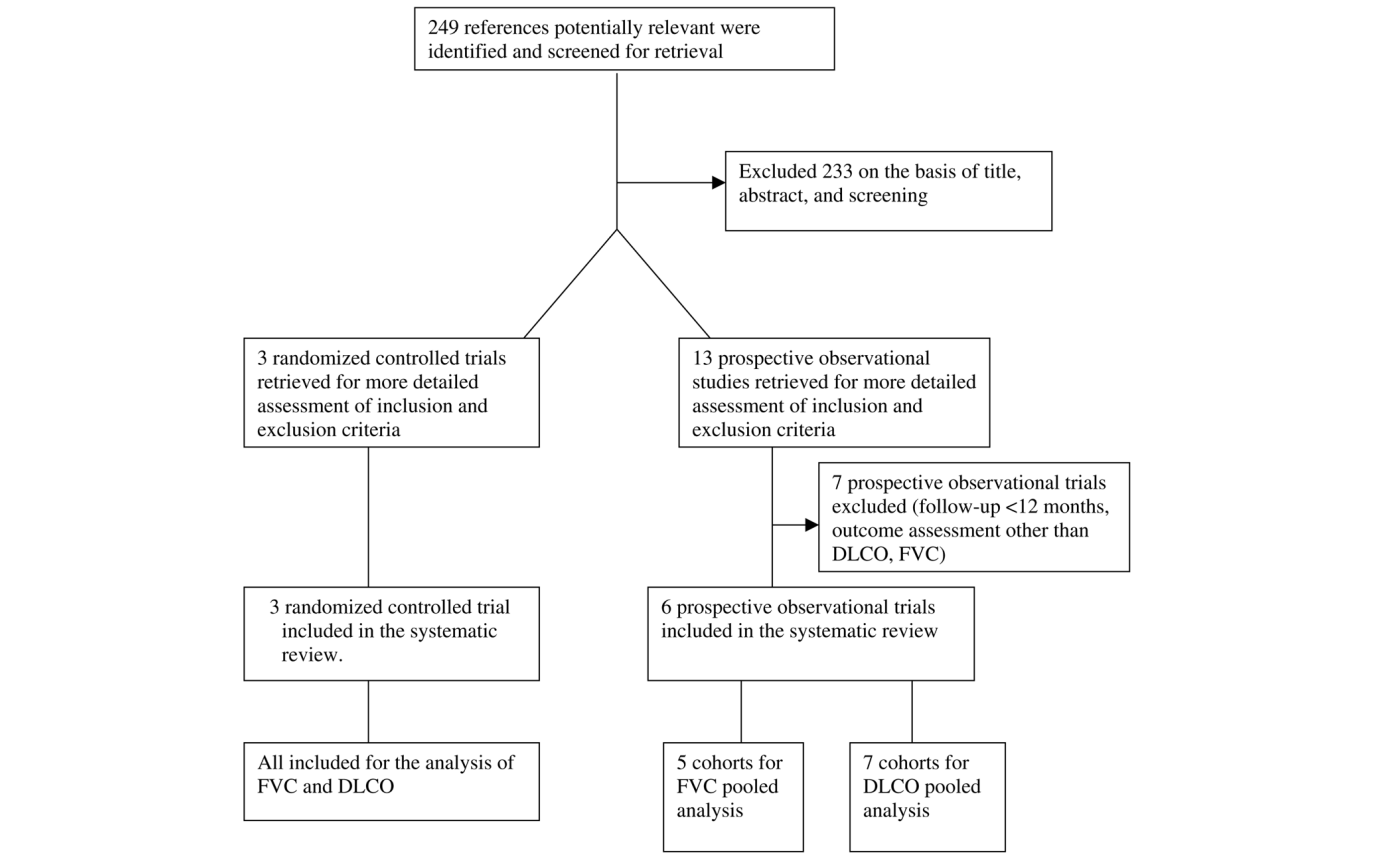
Meta-analysis study selection. DLCO, diffusing capacity for carbon monoxide; FVC, forced vital capacity.

Using the search key words, 249 references were identified and screened for retrieval. From this list, 47 potentially relevant full-text publications were selected. Of these, 31 full publications and 202 abstracts were excluded based on an unsuitable study population, the type of intervention or a lack of appropriate outcome assessment. A total of 16 studies (three randomized double-blind controlled studies and 13 observational studies) were then examined in detail. Five of the 13 observational studies were excluded due to inadequate length of follow-up (<12 months) and/or no information on the FVC and the DLCO as outcome assessments (Figure 1).

In the randomized controlled trials, both the FVC and the DLCO were evaluated at baseline and after 12 months in CYC treatment groups and in non-CYC treatment groups. In the observational study group, four our of six studies assessed the FVC at baseline and after 12 months of therapy, and five out of six studies assessed the DLCO at baseline and after 12 months. In one study, one-half of the patients received oral CYC and one-half of the patients were treated with intravenous CYC [12]. We analyzed the two cohorts of patients in this study separately. In addition, in this study the FVC and the DLCO were assessed in both cohorts at baseline and after 12 months [12], but it was not possible to calculate the standard error of the difference of FVC at 12 months since the authors reported only that this difference was not statistically meaningfully different (*P *> 0.05). In another study, 16 out of 28 patients were treated with high-dose corticosteroids (1 mg/kg/day for 4 weeks) and 12 out of 28 patients received lower dose corticosteroids (<10 mg/day) [16]. The FVC and the DLCO were assessed in both cohorts at baseline and after 12 months, and these cohorts were analyzed separately.

In the three randomized controlled clinical trials, patients and outcome assessors were masked to treatment allocation. In two trials, corticosteroid treatment was allowed [10,11]; in one of these, the control group was treated with azathioprine instead of placebo [11]. In the observational studies patients were allowed to use corticosteroid treatment with varying dose and tapering schemes. One study permitted enrollment of patients who had received treatment with disease-modifying drugs (D-penicillamine, cyclosporine, and combination of methotrexate, cyclosporine and azathioprine) but had discontinued their use at least 6 months prior to the study onset [15].

The included trials were somewhat heterogeneous in terms of the initial FVC and DLCO percentage predicted values (FVC percentage predicted value range = 51.4% to 90.4%, mean value = 70%; DLCO percentage predicted value range = 38.2% to 83.5%, mean value = 53.9%), the range of time from SSc-related ILD diagnosis (24 months to 7 years), the ILD stage assessment method (computed tomography scan, chest radiography or bronchoalveolar lavage), and the specific CYC treatment regimen. Table 2 presents the characteristics of all included randomized trials, and Table 3 presents the characteristics of observational trials. There was no clear evidence of heterogeneity by study quality, although this evaluation was limited by the small number of eligible studies and a lack of variability in quality across studies.

### Results of the meta-analysis

**Figure 2:**
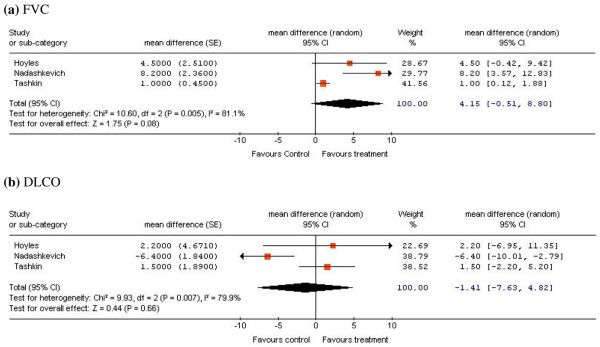
Forest plot of the overall meta-analysis results in the randomized clinical trials. Comparison of **(a) **the forced vital capacity (FVC) and **(b) **the diffusing capacity for carbon monoxide (DLCO) at 12 months for patients with scleroderma lung disease treated with cyclophosphamide versus a control group. See Table 2 for study details. RCT, randomized clinical trial; SE, standard error; CI, confidence interval; Chi^2^, chi-squared; df, degree of freedom; I^2^, *I*-squared; Z, *Z *value; Mean difference, weighted mean difference; Random, random-effects model.

In the randomized clinical trials, the FVC mean difference at 12 months between patients treated with CYC and patients treated with placebo or another immunosuppressant showed a positive trend in favor of the CYC group (mean difference = 4.15%), but did not reach statistical significance (95% confidence interval (CI) = -0.51 to 8.80; Figure 2a). The mean difference in the DLCO favored the control group (mean difference = -1.41%) but also did not reach statistical significance (95% CI = -7.63 to 4.82; Figure 2b).

**Figure 3:**
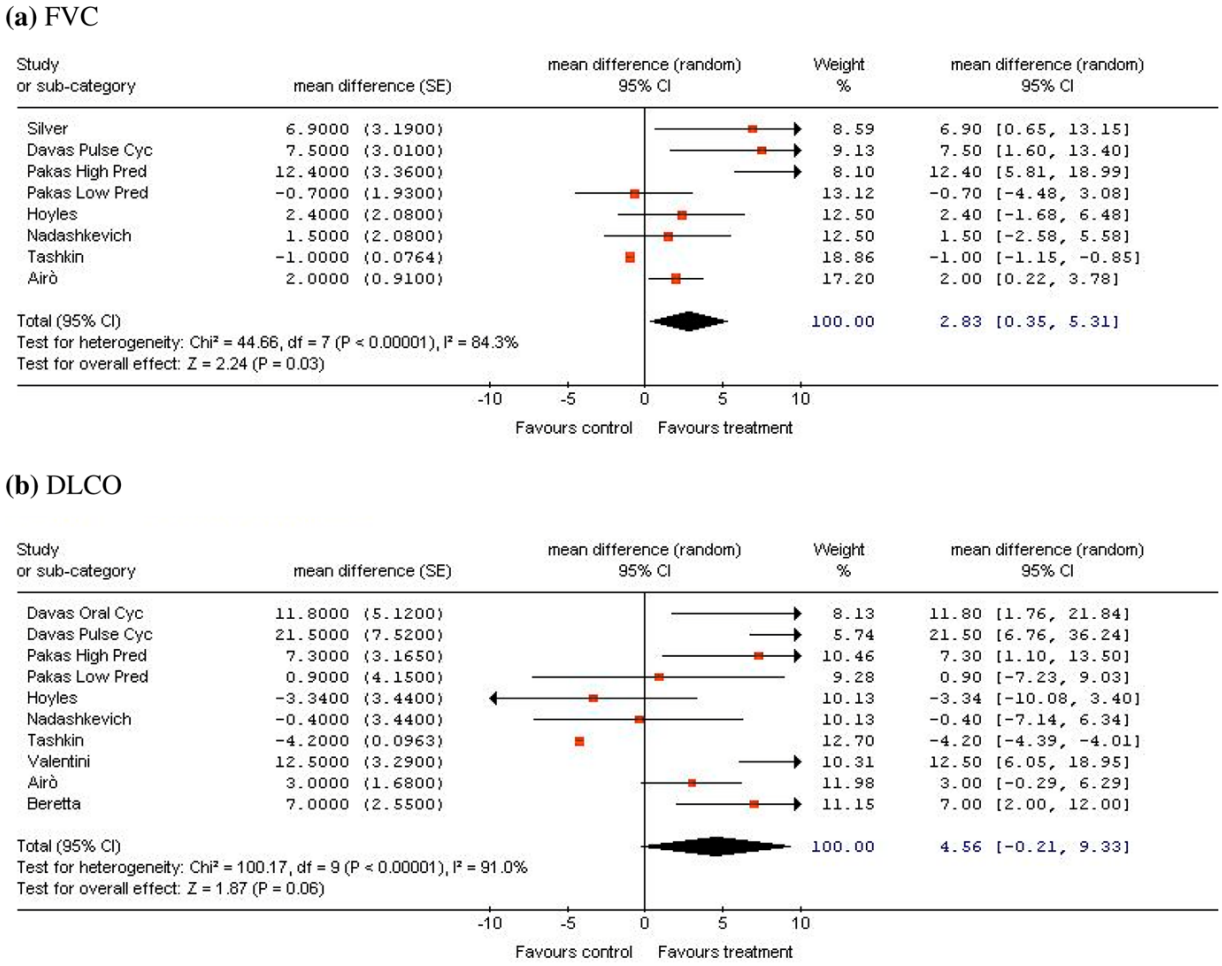
Forest plot of the overall meta-analysis results in randomized clinical trials and observational studies. Changes after 12 months of therapy versus baseline in **(a) **the forced vital capacity (FVC) and **(b) **the diffusing capacity for carbon monoxide (DLCO), pooled from the cyclophosphamide (CYC) arms of randomized clinical trials and observational studies. See Tables 2 and 3 for study details. SE, standard error; CI, confidence interval; Chi^2^, chi-squared; df, degree of freedom; I^2^, *I*-squared; Z, *Z *value; High Pred, high dose of prednisone; Low Pred, low dose of prednisone; Oral, oral administration; Pulse, intravenous administration; RCT, randomized clinical trial; Mean difference, weighted mean difference; Random, random-effects model.

In the observational studies, both the FVC and the DLCO predicted values after 12 months of therapy showed statistically significant improvement compared with baseline, with a mean difference of 4.73% (95% CI = 0.74 to 8.73) and 7.48% (95% CI = 3.64 to 11.32), respectively (data not shown). The pooled analysis of the treatment arms of the randomized clinical trials and of the observational studies suggested that both the FVC and DLCO predicted values improved after 12 months of therapy, with a mean difference of 2.83% (95% CI = 0.35 to 5.31) and 4.56% (95% CI = -0.21 to 9.33), respectively – although the latter change was not statistically significant (Figure 3a,b).

### Subgroup analysis of route of CYC administration

The change in FVC and DLCO values after 12 months of therapy did not differ between intravenous and oral CYC administration. Patients treated with oral CYC had a mean FVC change of 3% (95% CI = -0.88 to 6.87) and patients treated with intravenous CYC had a mean FVC change of 1.29% (95% CI = -1.76 to 4.33; test of interaction *P *= 0.086). Similarly, the group treated with oral CYC had a mean change in the DLCO of 6.38% (95% CI = 2.11 to 10.64), and patients treated with intravenous CYC had a mean change in the DLCO of 4.68% (95% CI = -0.31 to 9.67; test of interaction *P *= 0.6) (data not shown).

### Sensitivity analysis

We conducted a sensitivity analysis by introducing a range of standard error values for the mean difference between baseline and after 12 months of therapy from both the randomized and observational studies, since assumptions were necessary at this step as described in Materials and methods. No standard error within the range of those reported in the literature altered the results.

### Adverse events

We evaluated the relative risk of having adverse events in the CYC group compared with the control groups in the randomized studies; the open observational studies did not provide sufficient information to evaluate these adverse events. We considered as adverse events the occurrence of infections that required antibiotic therapy, hemorrhagic cystitis, hematuria and hospitalization. Only Tashkin and colleagues reported deaths: two (3%) in the CYC group and three (4%) in the placebo group [1]. The relative risk for adverse event occurrence did not differ among the treatment group and the control group (relative risk = 1.22, 95% CI = 0.75 to 2.00).

## Discussion

CYC is frequently recommended as treatment for scleroderma-related ILD. The results of the present meta-analysis suggest that patients with systemic sclerosis and ILD who are treated with CYC may experience a modest increase in the FVC and the DLCO after 12 months of therapy. Neither improvement in the FVC nor in the DLCO achieved clinical significance, however, as defined by an improvement of at least 10% of the predicted value of each measure [12,13]. The oral or intravenous administration routes of CYC did not influence the mean difference of the FVC or the DLCO after 12 months of therapy, and CYC treatment did not alter the risk of adverse events.

A change of more than 10% in the pulmonary function parameters evaluated in the studies reviewed in the present meta-analysis (or more) would have been considered clinically meaningful for the purposes of this study. Since this may or may not translate to clinically meaningful improvement, the conclusion that CYC treatment did not result in statistically meaningful improvement (rather than clinically meaningful improvement) could be justified.

To achieve a moderate Cohen effect size of 0.5 with 80% power, 64 patients per group would be required to detect a 10% difference in the FVC and DLCO predicted values [24]. This sample size was achieved by only one of the studies, which enrolled 73 patients per treatment arm but did not demonstrate an effect size of this magnitude [1]. The fact that the present meta-analysis, including 125 patients per group, did not achieve an effect size of this magnitude suggests again that the treatment approach is unlikely to be clinically meaningfully effective as assessed by these outcome measures.

Similar results were obtained in a retrospective study conducted in 103 SSc patients who were treated with oral CYC (1 to 2 mg/kg/day). The FVC and the DLCO improved by 4.3% and 1.0%, respectively, at 13 months of therapy compared with patients who were not treated with CYC [5]. Another retrospective study, however, suggested that patients treated with CYC had a larger increase in the FVC after 24 months of therapy (around 8% from baseline to 24 months of therapy) when compared with other treatment groups (prednisone, other immunosuppressant, D-penicillamine and no treatment) [25]. The DLCO demonstrated less consistent change [24]. The differences in results across these studies may be due in part to patient selection, as patients in these studies were not selected on the basis of ILD stage or progression.

Long-term CYC therapy may cause adverse events and treatment-related toxicity [3]. While reporting of adverse events in the included studies was limited, we found that the odds ratio of developing adverse events was similar among patients treated with CYC compared with patients in the non-CYC treatment groups (odds ratio = 1.29, 95% CI = 0.69 to 2.39). This lack of difference could also be due in part to the fact that Nadashkevich and colleagues permitted comparison between patients treated with azathioprine, which has a number of side effects in common with CYC, and patients treated with CYC [11]. Previous studies have reported no or very mild adverse events in patients with SSc-related ILD who were treated with CYC [26,27]. Other studies have reported bladder complications secondary to the drug in patients with SSc [28,29]. The adverse events counted in our nine studies included two cases of hemorrhagic cystitis [17] and several cases of hematuria – one case in Valentini and colleagues [18], two cases in Hoyles and colleagues [10] and nine cases in Tashkin and colleagues [1]; bladder cancer was not reported. A doubling of bladder cancer risk in Wegener's granulomatosis patients for every 10 g increase in the cumulative dose of CYC and an eightfold increased risk for treatment duration longer than 1 year has been reported [30]. Since the results of our meta-analysis are based on 12 months of follow-up they may not reflect adverse events developing over longer durations of treatment or follow-up.

Our study has additional limitations. The number of patients enrolled, the dose of CYC, concomitant corticosteroid use, the SSc-related ILD disease extent and SSc disease duration, and the comparator treatments varied across studies. For example, some evidence suggests that glucocorticoids may be effective in SSc-related ILD in certain situations [5,25,31-33]. There may be other factors contributing to heterogeneity unidentified by our review. The shortage of randomized controlled trials on this topic is a limitation, and larger randomized controlled trials are needed to better understand the role of CYC in the care of these patients. In our meta-analysis, two of the three greatest mean differences of the FVC after 12 months of therapy were achieved in observational studies using higher doses of corticosteroids [15,16], limiting our ability to draw a clear conclusion of beneficial effect of CYC alone. It is also possible that azathioprine has a beneficial treatment effect, which would reduce the magnitude of difference in benefit seen in comparison with CYC. A further limitation is that several studies, particularly the observational studies, had small numbers of patients.

## Conclusions

Based on available data, CYC treatment in patients with SSc-related ILD does not appear to result in clinically significant improvement of pulmonary function. Since none of the patients included in these studies were selected on the basis of progression of ILD or the time from the SSc-related ILD diagnosis, further randomized clinical studies are needed to evaluate whether CYC (or any) therapy might exert a beneficial effect in patients with worsening ILD. It is possible, for example, that patients treated sooner after diagnosis or at earlier stages of SSc-related ILD might have a better response to CYC treatment. Based on current understanding, however, SSc-related ILD will be only effectively addressed when better understanding of the immunopathophysiology of the disease is understood and when treatment options more effective than CYC become available.

## Abbreviations

ACR: American College of Rheumatology; AZA: azathioprine; BAL: bronchoalveolar lavage; CI: confidence interval; CREST: Calcinosis, Raynaud, ESophagitis, Sclerodactylia, Telangiectases; CT: computed tomography; CXR: chest radiography; CYC: cyclophosphamide; dcSSC: diffuse cutaneous systemic sclerosis; DLCO: diffusing lung capacity of carbon monoxide; EULAR: European League Against Rheumatism; FVC: forced vital capacity; HRTC: high resolution computed tomography; ILD: Interstitial Lung Disease; IV: intravenous; lcSSc: limited cutaneous systemic sclerosis; PFT: pulmonary function test; RR: relative risk; SE: standard error; SSC: systemic sclerosis; SSc-ILD: systemic sclerosis related interstitial lung disease; TLC: total lung capacity.

## Competing interests

The authors declare that they have no competing interests.

## Authors' contributions

CN conceived the study and participated in its design, coordination, data acquisition and analysis, and in manuscript preparation. CPW and ELM participated in the study design, data acquisition and analysis, and in manuscript preparation. PJE participated in data acquisition and in manuscript preparation. All authors read and approved the final manuscript.

## Supplementary Material

Additional file 1 Word table that reports the assessment of quality of randomized controlled trials. Click here for file

Additional file 2 Word table that reports the assessment of quality of observational studies. Click here for file
