# Supplementary material for: Effects of cyclophosphamide on pulmonary function in patients with scleroderma and interstitial lung disease: a systematic review and meta-analysis of randomized controlled trials and observational prospective cohort studies
Source: Arthritis Res Ther. 2008 Oct 20;10(5):R124. doi: 10.1186/ar2534 (PMC2592814; doi:10.1186/ar2534)
Supplement: Additional file 1 — Word table that reports the assessment of quality of randomized controlled trials. [file ar2534-S1.doc]

Additional data file 1. Assessment of quality of randomized controlled trials

| **Study RCT** | **Year** | **Randomization** | **Allocation** | **Blinding** | | | **Lost to follow up** | | **Intention to treat** | **Assessment of the outcome** | **Jadad Scale**  **score** |
| --- | --- | --- | --- | --- | --- | --- | --- | --- | --- | --- | --- |
|  |  |  |  | **Patients** | **Investigator** | **Data assessor** | **Cases (N-%)** | **Controls (N-%)** |  |  |  |
| **Tashkin DP et al. (1)** | 2006 | YES | YES | YES | YES | YES | 20 (25%) | 13 (16%) | YES | YES | 5 |
| **Hoyles RK et al. (8)** | 2006 | YES | YES | YES | YES | Not reported | 7( 32%) | 10 (43%) | YES | YES | 5 |
| **Nadashkevich O et al. (9)** | 2006 | YES | NO | NO | NO | NO | 0 | 0 | Not reported | YES | 2 |
