# Supplementary material for: Effects of cyclophosphamide on pulmonary function in patients with scleroderma and interstitial lung disease: a systematic review and meta-analysis of randomized controlled trials and observational prospective cohort studies
Source: Arthritis Res Ther. 2008 Oct 20;10(5):R124. doi: 10.1186/ar2534 (PMC2592814; doi:10.1186/ar2534)
Supplement: Additional file 2 — Word table that reports the assessment of quality of observational studies. [file ar2534-S2.doc]

Additional data file 2. Assessment of quality of observational studies

|  |  | **Selection** | | | **Comparability** | **Outcome** | | |  |
| --- | --- | --- | --- | --- | --- | --- | --- | --- | --- |
| **Study** | **Year** | 1  (star max 1) | 2  (star max 1) | 3  (star max 1) | 4  (star max 2) | 5  (star max 1) | 6  (star max 1) | 7  (star max 1) | Score  (max 8) |
| **Airo’ P et al (12)** | 2007 | 1 | 0 | 1 | 0 | 1 | 1 | 0 | 4 |
| **Beretta L et al (11)** | 2007 | 1 | 0 | 1 | 0 | 1 | 1 | 1 | 5 |
| **Davas EM et al (10)** | 1999 | 1 | 0 | 1 | 0 | 1 | 1 | 1 | 5 |
| **Pakas I et al (13)** | 2002 | 1 | 0 | 1 | 0 | 1 | 1 | 1 | 4 |
| **Silver RM et al (14)** | 1993 | 1 | 0 | 1 | 0 | 1 | 1 | 1 | 5 |
| **Valentini G et al (15)** | 2006 | 1 | 0 | 1 | 0 | 1 | 1 | 1 | 5 |
